# SSTP1, a Host Defense Peptide, Exploits the Immunomodulatory IL6 Pathway to Induce Apoptosis in Cancer Cells

**DOI:** 10.3389/fimmu.2021.740620

**Published:** 2021-11-19

**Authors:** Shyla Gopalakrishnan, Soumya Krishnan Uma, Gayathri Mohan, Amrutha Mohan, Geetha Shanmugam, Vineeth T. V. Kumar, Sreekumar J, Sivakumar K. Chandrika, Dileep Vasudevan, Sai Ravi Chandra Nori, Shijulal Nelson Sathi, Sanil George, Tessy Thomas Maliekal

**Affiliations:** ^1^ Cancer Research, Rajiv Gandhi Centre for Biotechnology, Thiruvananthapuram, India; ^2^ Interdisciplinary Biology, Rajiv Gandhi Centre for Biotechnology, Thiruvananthapuram, India; ^3^ Regional Centre for Biotechnology, Faridabad, India; ^4^ Manipal Academy of Higher Education, Manipal, India; ^5^ Statistics, Section of Extension and Social Science, The Indian Council of Agricultural Research (ICAR) Central Tuber Crops Research Institute, Thiruvananthapuram, India; ^6^ Genomics Core Facility, Rajiv Gandhi Centre for Biotechnology, Thiruvananthapuram, India; ^7^ Institute of Life Sciences, Bhubaneswar, India

**Keywords:** host defense peptides, temporins, immunoregulation, JNK/AP1 pathway, cancer, apoptosis, IL6 (IL-6) pathway

## Abstract

While the immunomodulatory pathways initiated in immune cells contribute to therapeutic response, their activation in cancer cells play a role in cancer progression. Also, many of the aberrantly expressed immunomodulators on cancer cells are considered as therapeutic targets. Here, we introduce host defense peptide (HDP), a known immuomodulator, as a therapeutic agent to target them. The cationic host defense peptides (HDPs), an integral part of the innate immune system, possess membranolytic activity, which imparts antimicrobial and antitumor efficacy to it. They act as immunomodulators by activating the immune cells. Though their antimicrobial function has been recently reassigned to immunoregulation, their antitumor activity is still attributed to its membranolytic activity. This membrane pore formation ability, which is proportional to the concentration of the peptide, also leads to side effects like hemolysis, limiting their therapeutic application. So, despite the identification of a variety of anticancer HDPs, their clinical utility is limited. Though HDPs are shown to exert the immunomodulatory activity through specific membrane targets on immune cells, their targets on cancer cells are unknown. We show that SSTP1, a novel HDP identified by shotgun cloning, binds to the active IL6/IL6Rα/gp130 complex on cancer cells, rearranging the active site residues. In contrast to the IL6 blockers inhibiting JAK/STAT activity, SSTP1 shifts the proliferative IL6/JAK/STAT signaling to the apoptotic IL6/JNK/AP1 pathway. In IL6Rα-overexpressing cancer cells, SSTP1 induces apoptosis at low concentration through JNK pathway, without causing significant membrane disruption. We highlight the importance of immunomodulatory pathways in cancer apoptosis, apart from its established role in immune cell regulation and cancer cell proliferation. Our study suggests that identification of the membrane targets for the promising anticancer HDPs might lead to the identification of new drugs for targeted therapy.

## Introduction

In the tumor microenvironment, the immunomodulators expressed on immune cells and cancer cells play a role in the progression of the disease. The immunoregulatory pathways involved in immune evasion, and their relevance in immunotherapy have been well-explored in the past decade (1, 2). Moreover, some of the immune regulatory molecules, mainly cytokines, have been reported to be associated with cancer properties including proliferation, invasiveness and aggressiveness (3–6). Consistent with their role in cancer progression, immunomodulatory pathways, like IL6 pathway, are identified as therapeutic targets in different cancers (7, 8).

Host defense peptides (HDPs), thought initially as antimicrobial peptides, are now considered as immunomodulators because of their multi-faceted roles in the host’s innate and adaptive immunity. They kill bacteria by disrupting the bacterial membrane *via* pore formation in the lipid membrane, thinning of the membrane bilayer, membrane dissolution, or lipid-peptide domain formation (9). Certain immunomodulatory activities are also reported for HDPs, like the stimulation of cytokines and chemotaxis for leukocytes (10). Apart from these functions, some of the human HDPs, like Cathelicidin, α-Defensins and β-Defensins are shown to modulate tumor properties (11). Some of the evidences from Cathelicidin null mice reinforce the role of HDPs in tumor suppression (12). Over-expression of a Cathelicidin, LL-37, suppresses tumorigenesis in colon and gastric cancer cells (13). LL-37 interacts with *N*-formyl peptide receptor like 1 (FPRL1) on immune regulatory cells (14, 15), increasing their cytotoxic effects on cancer cells, leading to tumor suppression (13). In contrast, LL-37 promotes tumor progression in some contexts, depending on its binding to FPR2 and P2X_7_R in pancreatic cancer (16) or IGF-1R in breast cancer (13). Nevertheless, the mechanism by which LL-37 induces apoptosis in cancer cells of different origin is not clearly understood (11, 17). In parallel, several other HDPs of nonhuman origin also induce apoptosis in cancer cells independent of the innate immune regulatory mechanisms (18).

HDPs isolated from amphibians and marine organisms have been widely investigated for their antitumor activity based on their antimicrobial activity. Since cancer cell membrane has a high negative charge on the outer surface due to aberrant expression of anionic molecules, majority of the reports show the basis of antitumor property as membranolytic activity, similar to the bacterial membrane pore formation (18). The non-specific membranolytic activity limits the clinical translation of antitumor HDPs, as it leads to hemolysis and other side effects (9–11) However, the induction of apoptosis might depend on mechanisms other than the regulation of innate immunity and membranolytic activity, involving specific cell signaling (18). Although certain bioactive peptides induce apoptosis depending on the modulation of ROS production and the activation of the mitochondrial pathway (19), the upstream signaling pathways activated are not yet identified (20, 21).

Recalling the relevance of FPRL1 for LL-37, it can be speculated that the upstream signaling pathway initiated by HDPs could be one of the immunomodulatory pathways, which leads to apoptosis. Here, we attempted to elucidate the mechanism by which one of the HDPs, identified from the skin secretion of an indigenous frog, induces apoptosis in oral cancer cells. The present study suggests that the immunomodulatory mechanism exerted by HDPs on cancer cells leads to cell death.

## Materials and Methods

### Cells and Reagents Used

HSC-4 and RCB1015 cells were collected from RIKEN BRC *Cell* Bank, Japan. CaSki, MDA-MB-231, SiHa, HeLa and A-375 were obtained from RGCB cell repository. The following antibodies for IL6Rα (clone 2B2.3, Clone H.7),DSG-3 (clone5H10), βTubulin (clone37), α5 tubulin, β- actin (Clone C4), GRPR (clone H50), Shh (cloneN-19), DLL (clone C20) PARP-1 (clone B10), STAT 3(clone F2), JNK 1 (clone C-17), c-JUN (clone D) and Rab5C (C-13) were purchased from Santacruz Biotechnology Inc, USA. Antibodies for Caspase 9 (clone C9), Caspase 8 (clone1C12), Caspase 3, Caspase 7 (clone D2Q3L), phospho-STAT3 (clone 6E4), phospho-STAT3 (clone D3A7), phospho-JNK, phospho-c-JUN, STAT1 (clone D4Y6Z), phospho-STAT1 (clone 58D6), phospho-MAPK1/2 (clone D13.14.4E), and phospho-AKT, were purchased from Cell Signaling Technology, USA. Secondary antibodies used for western blot are Peroxidase conjugated AffiniPure Donkey Anti Mouse, AffiniPure Donkey Anti Goat and AffiniPure Donkey Anti-Rabbit Secondary Antibodies (Jakson ImmunoResearch Laboratories Inc.). Donkey Anti-Goat Alexa Fluor-568, Donkey Anti-Mouse Alexa Fluor-568, Donkey Anti-Rabbit Alexa Fluor-568, Donkey Anti-Goat Alexa Fluor-680 and Streptavidin Alexa Flour-488 (Molecular Probes, Life Technologies) were used for immunofluorescence.

SSTP1 (FLPLLISALTSLFPKLGK) and SSTP2 (FLPRRISARTSLFPKRGK–NH_2_) were synthesized from SynPeptide Co. Ltd, Shanghai, China. Peptides were dissolved in Milli-Q water and aliquoted. Short term storage was at 4 °C and the other aliquots were stored at -20 °C. Peptides were labeled with D-Biotin Succinimidyl ester (Life Technologies) or Alexa Fluor^®^ 488 5-SDP ester (Life Technologies). 3xAP1pGL3 was a gift from Alexander Dent (Addgene plasmid#40342; http://n2t.net/addgene:40342; RRID : Addgene 40342). MitoTracker Deep Red (Thermo Fisher Scientific) was used for imaging. S-Ruxolitinib (Selleckchem), SP600125 (InvivoGen) and LMT- 28 (Sigma Aldrich) were the inhibitors used for the study. Dynabeads M-280 Streptavidin (Invitrogen) was used for pull-down assay.

### Labeling of Peptides With D-Biotin or Alexa Fluor 488

Peptide was labeled with D-Biotin Succinimidyl ester or Alexa Fluor^®^ 488 5-SDP ester in a 5:1 ratio in bicarbonate buffer (pH=8.3). The mixture was stirred continuously at room temperature for 30 minutes, after which different fractions of labeled peptide were eluted through Sephadex G-25 column with Milli-Q water. The fractions with higher concentrations of peptide (Absorbance at 280) and dye (Absorbance at 350 or 488nm respectively for biotin or Alexaflour 488 dye) together were used.

### Screening of cDNA Encoding Peptides Through Shotgun Cloning

cDNA library constructed from frog skin secretions (10) was subjected to RACE amplification with a sense (5’ATGTTCACCTTGAAGAAT) and anti-sense primer (5’- AGATGATTTCCAATTCCAT-3’) Purified amplicons were cloned into pGEMT-easy vector (Promega) and positive clones were amplified and sequenced (ABIDNA sequencer 3730). The nucleotide sequences were translated to amino acid sequence with EMBOSS Transeq (http://www.ebi.ac.uk/Tools/st/emboss_transeq/) and were subjected to homology searches using NCBI BLAST (http://w.w.w.ncbi.nlm.nih.gov). Antimicrobial peptide database (APD, http://aps.unmc.edu/AP/main.php) was used for peptide prediction. Physical parameters were computed using ProtParam (http://expasy.org/tools/protparam.html).

### MTT and IC- 50 Calculation

Concentration- and time-dependent effects on cell growth inhibition were measured using the MTT [3-(4,5-dimethylthiazol-2-yl)−2,5-diphenyltetrazolium bromide] assay. 5000 cells/well were seeded in 96-well plate and were treated with peptides for 48h. Cells were incubated with MTT (1.25 mg/ml) for 2 hours at 37°C. The blue formazan crystals were solubilized in isopropanol and read at 595 nm.

### Annexin/PI Staining

The cells were stained with Annexin V apoptosis detection kit (Santacruz Biotechnology, Inc), following the instructions of manufacturer and analyzed using BD FACS AriaII.

### Western Blotting

Western blotting was performed as reported earlier (22). Total proteins extracted from cells were resolved on SDS-PAGE, transferred onto nitrocellulose membranes, which were incubated with corresponding primary antibody followed by horseradish peroxidase-linked secondary antibody. The images were developed using Clarity western ECL substrate (Bio-Rad) and images were captured on X-Ray sheet. Densitometric Analysis was performed using software ImageJ 1.52v. The standard deviation was calculated for biological duplicates.

### Immunofluorescence Staining and Confocal Microscopy

HSC-4 cells were grown overnight on confocal dish. Immunofuorescence was performed as described (22). The images were acquired in Nikon A1R LSCM or Leica SP8 Wll Confocal Microscope.

### Co-localization Studies

The images were acquired in Nikon A1R LSCM confocal microscope with 60× objective, oil immersion and the analysis was performed using NIS-Elements AR 4.00.04. For the co-existence of SSTP1 and transferrin, SSTP1 was labeled with Alexa Fluor-488 and transferrin with Alexa Fluor-568. The imaging was performed with a pinhole size of 39.7 µm giving an optical resolution of 0.10 µm for Alexa Fluor-488 and 0.12 µm for Alexa Fluor-568. The Mander’s overlap was calculated. For analyzing the physical interaction of SSTP1 and IL6Rα, Biotin-labeled SSTP1 (10 µM) was added to cell and incubated for 30 minutes. Cells were washed, fixed, blocked with 2% FBS and probed for IL6Rα using antibody for extracellular domain. The signals were visualized using streptavidin-488 and anti-mouse Alexa Fluor-680. The imaging was performed with a pinhole size of 43.2 µm giving an optical resolution of 0.10 µm for Alexa Fluor-488 and 0.13 µm for Alexa Fluor-680. The co-localization indicating a physical interaction was calculated as Pearson’s co-efficient. To study the endosomal co-localization of SSTP1 and IL6Rα, the experiment was performed as before. Streptavidin-Alexa Fluor-488, anti-mouse Alexa Fluor-568 and anti-goat Alexa Fluor-680 were used for visualization. The imaging was performed with a pinhole size of 43.2 µm giving an optical resolution of 0.10 µm for Alexa Fluor-488, 0.12 µm for Alexa Fluor-568 and 0.13 µm for Alexa Fluor-680. Mander’s overlap was calculated to analyze the co-existence. In all cases, single staining was performed to rule out the bleed through.

### FRET Analysis

For the FRET analysis, Alexa Fluor-488 and Alexa Fluor-555 was used as the FRET pair. Cells were treated with biotin-labeled SSTP1 (10 µM) for 30 minutes, unbound peptides were washed off and cells were fixed and stained for IL6Rα. To visualize staining streptavidin-Alexa Fluor-488 and anti-mouse Alexa Fluor-555 were used. The images were acquired using Olympus FV3006 in FRET mode. The image analysis was performed using FV31S-SW software.

### RNA Isolation, Library Preparation and Sequencing

RNA was isolated from the cells using RNeasy mini kit (Qiagen). mRNA-Sequencing libraries were prepared using NEBNextR Ultra™ II RNA Library PrepKit for IlluminaR (NEB) following the manufacturer’s instructions. The sequence data quality was checked using FastQC and MultiQC software. The QC passed reads were mapped onto indexed Human reference genome (GRCh38.p7) using TopHat2 and were annotated with Human GTF (GRCh38) downloaded from GENCODE. Transcripts were assembled using Cufflinks. The assembled transcripts of the samples were merged together using the Cuffmerge. Cuffcompare was used to compare the assembled transcripts with GENCODE, annotation. Differential gene expression analysis was carried out using Cuffdiff. The genes that showed significant differential expressions were used for pathway enrichment analysis by WebGestalt.

### RT-PCR and qRT-PCR

Total RNA was extracted and reverse-transcribed using Maxima Reverse transcriptase (Thermo Scientific). RT-PCR or qRT-PCR was done to assess gene expression with gene-specific qRT-PCR primers (**Supplementary Table 4**). RT-PCR was done using Master cycler Gradient PCR (Eppendorf). qRT-PCR reactions were done in 7500 Real-time PCR System (Applied Biosystems) using the Maxima SYBR green qPCR master mix; (Thermo Fisher).

### Pull-Down Assay

HSC-4 cells were lysed with immunoprecipitation buffer [0.025M Tris, 0.15 M Sodium chloride, 0.001 M EDTA, 0.5% (v/v) NP40, 0.5% (v/v) Triton X 100, 5%glycerol (v/v), 1× protease inhibitor, pH 7.4]. For affinity pull-down, 10 μmols of biotin-labelled peptides were incubated with cell lysate containing 1 mg of protein, for 1 hour at 4°C followed by overnight incubation with streptavidin Dynabeads. After washes, bound peptide-protein complexes were separated from the beads by boiling in 5× dye, resolved on 10% SDS-PAGE and were used for western blot analysis.

### Docking and Dynamic Simulation

SSTP1 and SSTP2 structures were modeled individually using the PEP-FOLD3 peptide structure prediction server (23) by biased model approach with default settings, using the structure of Temporin-1 Ta (PDB id: 2MAA). The best models of SSTP1 and SSTP2, as suggested by the server were chosen. Docking of SSTP1 and SSTP2 were done with IL6/IL6Rα/gp130 complex (PDB No.1P9M) using ClusPro and docking scores were calculated. Structures with highest scores were further used for simulation studies. The structures obtained from the molecular docking were subjected to molecular dynamics simulation using GROMACS version 5.0.4 with all-atom/OPLS-AA force field. The docked models were solvated in a periodic cubic box containing flexible, simple point charge (SPC) water molecules and neutralized with Na+ and Cl- counter ions replacing the water molecules. The particle mesh Ewald method was used for electrostatic interactions of the simulation system periodic boundary conditions with grid dimensions of 1.0 Å and a 12.5 Å cut-off for Van der Waals interaction. The prepared systems underwent minimization (5000 steps of steepest descent and 5000 steps of conjugated gradient) and further subjected to equilibration at a temperature 300 K and a pressure 1 bar for a total of 10 ns. Finally, the systems were subjected to MD simulations for 100 ns, and the atomic coordinates were recorded every 10 ps for further analysis. Comparative structural deviations in systems were analysed with the coordinates of the vectors generated during MD simulations after obtaining hierarchal MD clusters. Interface area (Å2) and hydrogen-bonds were calculated using the PISA server.

### Reporter Assay

HSC-4 cells (50,000 cells/well) were seeded in a 24-well plate, and 3×AP1pGL3 (24) and Renilla luciferase plasmids were transfected in 10:1 ratio using Lipofecatmine2000 (GIBCO). Cells were treated with the peptides, and cell lysates were prepared to perform luciferase assay using Dual Luciferase Reporter Assay system (Promega), following manufacturer’s instruction.

### Generation of IL6Rα-Knocked-Down Cells

HSC-4 cells were infected with lentiviral particles containing either non-targeted control shRNA or a pool of three shRNAs against human IL6Rα. The stable cells were selected using puromycin.

### Hemolytic Assay

The protocols of the assays with human samples were approved by the human ethics committee of Rajiv Gandhi Centre for Biotechnology, Thiruvananthapuram, (IHEC/01/2017/20). Blood samples were collected from volunteers after getting the informed consent. Hemolytic assays of SSTP1 was carried out as described (25). Briefly, fresh human RBCs were re-suspended in PBS to make 4% (v/v) solution and exposed to serially diluted peptides in PBS at 37°C. After 1 h, the amount of hemoglobin released was estimated by measuring the absorbance of the supernatant at 415 nm. Hemolysis with Triton X-100 was used as the positive control.

### Statistical Analyses

IC_50_ value for HSC-4 cells and MDA-MB-231cells were calculated with Dose-response model. Analysis of dose-response data is made available through the ‘drc’ package in R environment for statistical computing. The “drm” function in the package “drc” is a general model fitting function for analysis of concentration/dose/time-effect/response data and the four parameter a log-logistic model was fitted for estimating the slope, lower, upper and IC_50_ values. All the other data analyses were performed using GraphPad Prism 8.4.3. The line graphs were prepared using non-linear curve fit–four parameters. The statistical significance between two groups was analyzed using Students paired two-tailed t-test.

## Results

### Identification of Antitumor Peptides From the Skin Secretion of *Indosylvirana aurantiaca*

Skin secretion from *Indosylvirana aurantiaca* was used to construct a cDNA library by shotgun cloning (25). The 14 mature peptides identified from the frog’s skin secretion (**Supplementary Table 1**) were chemically synthesized to check the antitumor activity. One of the peptides, Temporin 1IDau1, was chosen for further analysis due to its low IC_50_ value. This peptide is referred to as SSTP1 in our studies. The physicochemical properties of SSTP1 are listed in **Supplementary Table 2**. The helical wheel projection of SSTP1 showed a spatial separation of hydrophilic residues and hydrophobic residues imparting amphipathicity (**Supplementary Figure 1**). Though there were several hydrophobic residues, we modified the sequence by replacing four of the leucines with Arginines to generate a control peptide, SSTP2, with less helicity and amphipathicity. Thus, the *in silico* analysis predicted SSTP2 to be a negative control for the potent antitumor peptide, SSTP1. The peptides were chemically synthesized and its purity was confirmed by HPLC profile and ESI/MS analysis (**Supplementary Figure 2**).

### SSTP1 Induces Apoptosis in Oral Cancer Cells

**Figure 1:**
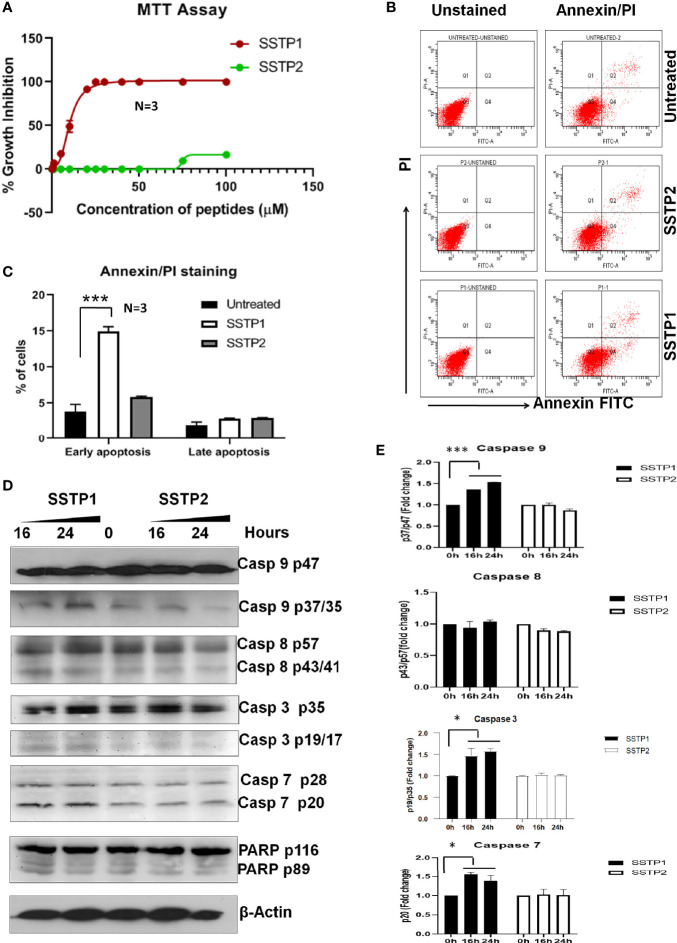
SSTP1 induces apoptosis in oral cancer cells **(A)** HSC-4 cells were treated with peptides SSTP1 and SSTP2 at different concentrations in 0.5% DMEM for 48 h, and MTT assay was performed to calculate % growth inhibition. The graph was plotted using GraphPad Prism8.2.1using non-linear four parameter curve fit. The error bars indicate SEM of three biological replicates **(B)** HSC-4 cells were treated with 10 μM SSTP1 and SSTP2 for 22h and FACS analysis was performed after annexin/PI staining. **(C)**The quantified populations are represented in the graph. The error bars indicate standard deviation of triplicates. **(D)** The HSC-4 cells treated with 10 μM SSTP1 or SSTP2 for indicated time intervals were used for western blot analyses **(E)** Densitometric analysis was done for the bands, and fold change based on 0min was calculated. The error bars indicate standard deviation of biological duplicates. *p < .05, ***p < .001.

In HSC-4 cells, SSTP1 induces growth inhibition with an IC_50_ value of 10.22 μM, while SSTP2 has negligible cytotoxicity (**Figure 1A**). We performed Annexin/PI staining, and the population with Annexin alone (early apoptotic), Annexin/PI dual stained (late apoptotic), and PI alone (dead cells) were quantified by FACS. We observed a 3-fold increase in the early apoptotic population upon SSTP1 treatment, from 3.8% to 14. 9%, while there was no significant change in the late apoptotic population (1.9% increased to 2.9%) and dead cell population (less than 0.1% in all cases) (**Figures 1B, C**). Further analysis showed the active cleavage of PARP, Caspase 3, 7, and 9, while Caspase 8 was unaffected by SSTP1 treatment. The cells treated with SSTP2 neither showed an increase in apoptotic population nor activation of any of the Caspases (**Figures 1D, E**). As all these evidences suggested the involvement of mitochondrial pathway of apoptosis, we explored the upstream regulators of this pathway.

### Induction of Apoptosis by SSTP1 Involves a Non-Membranolytic Activity

**Figure 2:**
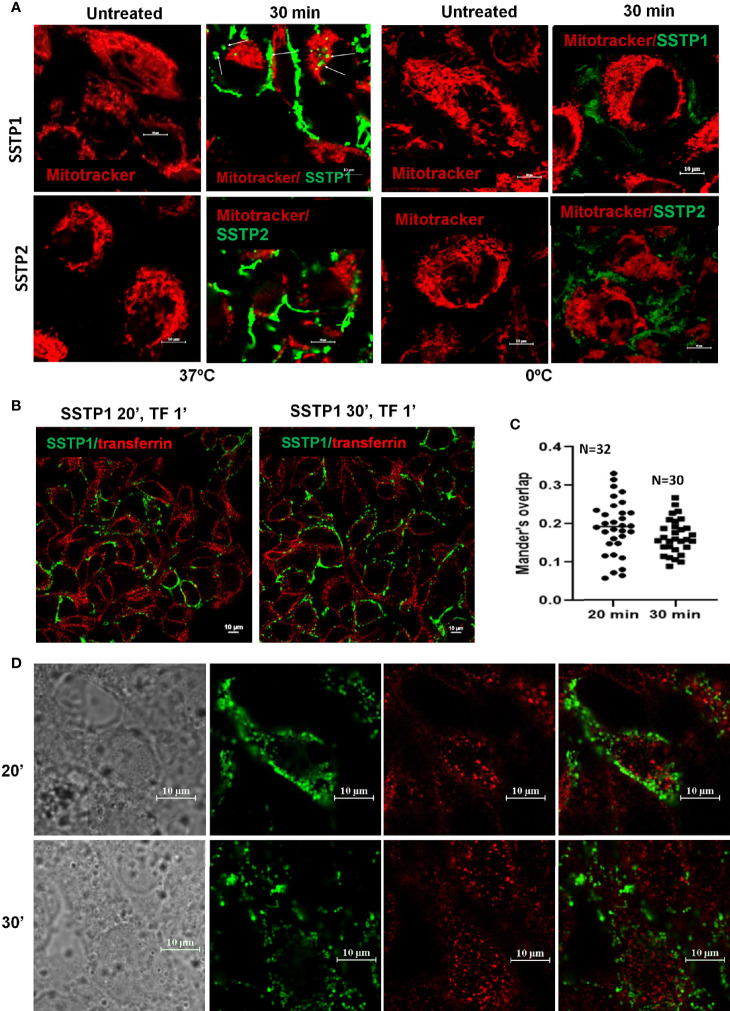
SSTP1 is internalized by active uptake. **(A)** Live HSC-4 cells were incubated with MitoTracker Deep Red and incubated for 10 min. Excess dye was washed off, and then the respective peptides were added, which were labeled with Alexa Fluor-488, and incubated either on ice or at 37°C. The unbound peptide was washed off and the images were acquired at the indicated time intervals. The arrowmarks indicate the internalized peptide. Magnified images along with DIC are provided in **Supplementary Figure 3**. **(B)** The cells were treated with Alexa Fluor-488-labelled SSTP1 for indicated time and Alexa Fluor-568-labelled transferrin was added and incubated for one minute. The cells were washed, fixed and imaged. **(C)** The extent of co-occurrence for panel B was measured and plotted as Manderson’s overlap. The median value is shown in the graph. **(D)** The high magnification representative images of **(B)** The scale bars represent 10 μm.

Based on our initial standardization, we have seen that the peptide binds to the membrane by 30 minutes and it stays on the membrane till 4 hours. The pattern of SSTP1 was speckled than diffused (**Supplementary Figure 3A**). We observed some speckles inside the cell membrane as well (**Figure 2A**, **Supplementary Figure 3B**). The later experiments were conducted at 30 minutes. Though at a lower rate, SSTP2 was also binding to the membrane and internalizing. When we repeated the experiments on ice, where cells were not physiologically active, SSTP1 and SSTP2 were not appreciably bound or internalized as observed at 37°C, (**Figure 2A**). Since these results suggested the involvement of a receptor-mediated internalization for SSTP1, we decided to check whether it is clathrin-dependent mechanism, using labeled transferrin (**Figures 2B–D**). Transferrin is used as a marker for clathrin-mediated endocytosis and the intracellular trafficking of this molecule is well-established. It is known that the molecule enters clathrin-mediated early endosomes within one minute (26, 27). As evident from **Figure 2D**, transferrin and SSTP1 entered the cytoplasm, but they did not co-exist. SSTP1 did not internalized along with transferrin, as suggested by the negligible Mander’s overlap, the entry of SSTP1 is probably through receptor-dependent clathrin-independent endocytosis. We further investigated the genes and pathways activated by SSTP1 treatment, which might give insight to its membrane receptors.

### RNA-Seq Analysis Suggests the Involvement of the IL6/IL6R Pathway in SSTP1-Induced Apoptosis

**Figure 3:**
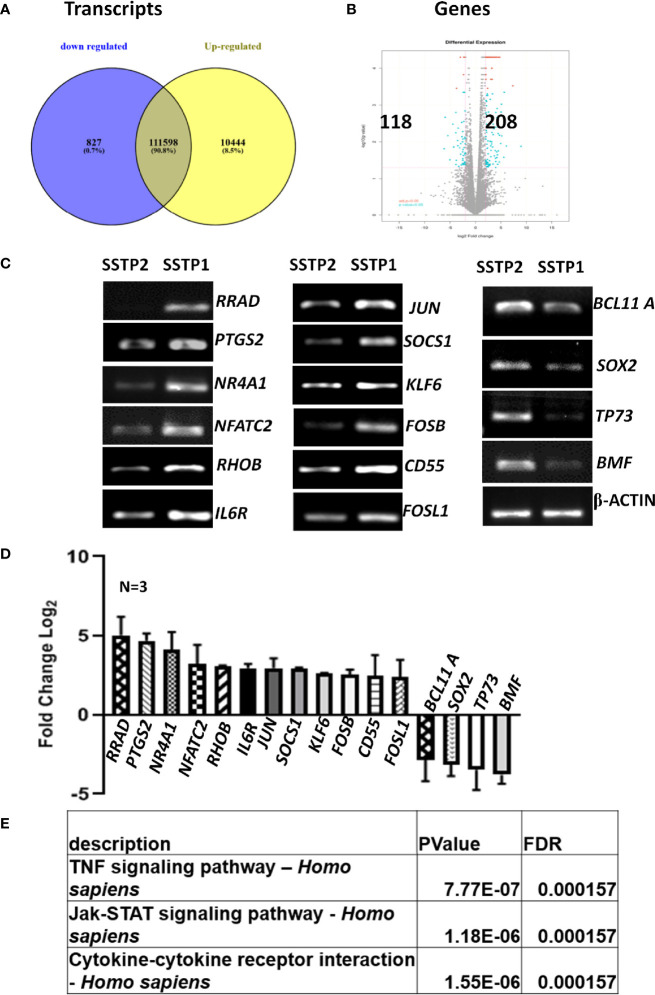
RNA-Seq analysis suggests the activation of cytokine signaling pathway upon SSTP1 treatment. **(A)** HSC-4 cells were treated with 10 μM SSTP1 and SSTP2 for 3h, and total RNA was isolated to perform RNA-Seq analysis. The Venn diagram represents the differential expression of transcripts upon SSTP1 treatment compared to SSTP2-treatment. **(B)** Volcano plot showing differential expression profile of genes showing significant up regulation and down regulations on treatment with SSTP1 with respect to control peptide SSTP2. Red dots indicate absolute log2 fold change≥2 and FDR- adjusted p value ≤ 0.05 and blue dots indicate log2 fold change≥2 and p value ≤ 0.05. **(C, D)** The results were confirmed by RT-PCR and qPCR for the indicated molecules. **(E)** A pathway enrichment analysis was performed based on the differential gene expression analysis, and top 3 pathways that were regulated are tabulated.

**Figure 4:**
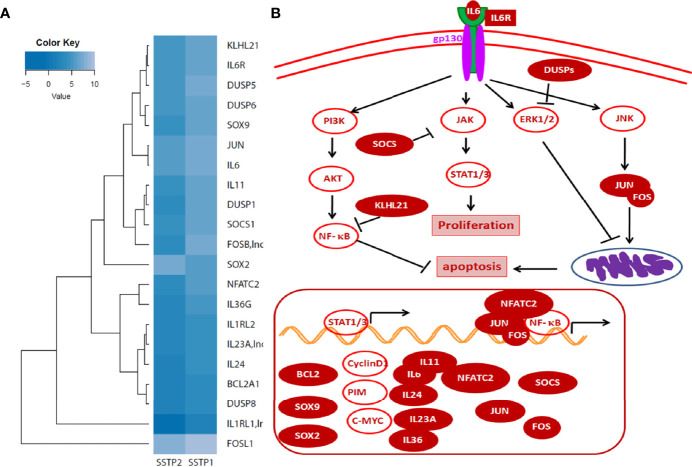
Cytokine signaling pathway involved in SSTP1 response **(A)** STAT/.JAK, JNK/AP1 MAPK1/2, and AKT pathways are reported to be downstream of Cytokine signaling. Heat map showing differential expression of molecules involved in cytokine signaling are shown. **(B)** Schematic diagram of cytokine signaling pathway in SSTP1 response.

An RNA-Seq on the Illumina platform was performed to identify the target genes of SSTP1, using cells treated with SSTP1 in comparison to SSTP2. Out of the 34 to 37 million reads obtained, 31 to 34 million reads were mapped onto the indexed Human reference genome GRCh38.p7. The differential gene expression analysis identified 208 up-regulated genes and 118 down-regulated genes (**Figures 3A, B**). The RNA-Seq results were validated by 12 up-regulated genes and four down-regulated genes by RT-PCR and q-PCR (**Figures 3C, D**). Several of the differentially expressed genes were related to apoptosis, (**Supplementary Table 3**), and the most significant pathway regulated was cytokine signaling (**Figure 3E**). There were interleukins and their receptors along with other regulators of cytokine signaling (**Figure 4A**). **Figure 4B** and **Supplementary Figure 4** represent a possible cytokine signaling pathway that leads to apoptosis upon SSTP1-treatment. Molecules like *DUSPs* and *SOCS1*, which regulate STAT and MAPK functions, respectively, were up-regulated by SSTP1 treatment. There was a notable up-regulation of AP1 subunits, *JUN* and *FOS*. Since the ligand, receptor and intermediates for the IL6/IL6R pathway were up-regulated, we focused on this pathway. Yet, the pro-tumorigenic targets of the JAK/STAT pathway, like *CyclinD1, cMyc* and *PIM* were not regulated.

### SSTP1 Binds to IL6Rα on The Cell Membrane

**Figure 5:**
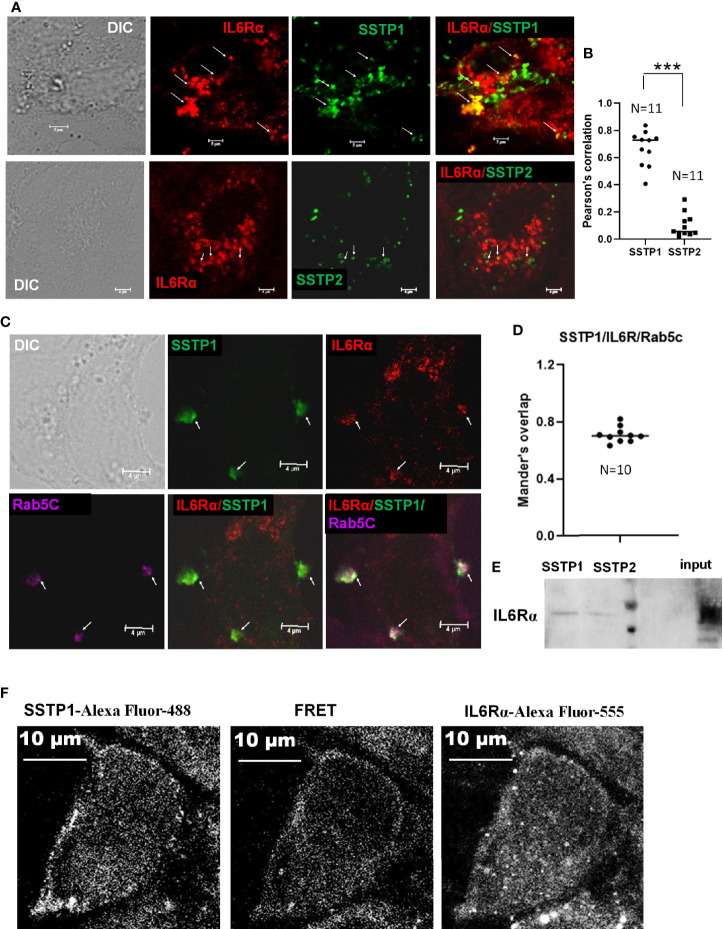
SSTP1 interacts with IL6Rα and mediate the uptake of SSTP1 **(A)** HSC-4 cells were treated with Biotin labeled-SSTP1 or SSTP2 (10 μM) for 30 minutes and then fixed and probed without permeabilization using mouse IL6Rα (2B2.3). **(B)** The co-localization of SSTP1 or SSTP2 to IL6Rα was analyzed for 11 cells, and the Pearson’s correlation was plotted. **(C)** Cells were treated as in panel A and fixed and permeabilized to probe for IL6Rα, SSTP1 and Rab5C. The arrow mark indicates co-localization. The scale bars represent 4 μm **(D)** The Mander’s overlap in SSTP1 foci were quantified for 10 cells. The average value for each cell is plotted **(E)** Pull-down assay using SSTP1-biotin or SSTP2-biotin and probed for IL6Rα. **(F)** FRET analysis to show the SSTP1 binding to IL6Rα as described in Methods. The scale bars represent 10 μm for panel **(A, F)**. ***p < .001.

To check whether SSTP1 activates the IL6/IL6R pathway by binding to IL6Rα, we checked the co-localization of SSTP1 and IL6Rα on cell membrane. We observed several foci on the cell surface with co-localization of SSTP1 and IL6Rα, while co-localization of SSTP2 to IL6Rα was rare as shown by the Pearson’s coefficient (**Figures 5A, B**). In order to rule out a nonspecific interaction of SSTP1 to a random surface receptor, we performed a co-immunofluorescence analysis of SSTP1 with several surface molecules like tubulin α5, β-tubulin, DSG3, DLL1 and GRPR, which did not show co-localization with the peptide (**Supplementary Figure 3**). To visualize the IL6R-dependent SSTP1 endosomal up-take, we used an early endosome marker, Rab5C. We observed the co-existence of SSTP1 and IL6Rα within early endosomes, marked by Rab5C (**Figures 5C, D**). The physical interaction of SSTP1 with IL6Rα was stronger than that of SSTP2, as revealed by the pull-down assay using biotin-labeled peptides (**Figure 5E**). The physical interaction of SSTP1 and IL6Rα was also ascertained using FRET analysis as described under *Materials and Methods* (**Figure 5F**).

### *In Silico* Analyses Suggest That SSTP1 and SSTP2 Exert Differential Effects on Active Sites of IL6/IL6Rα/gp130 Complex

**Figure 6:**
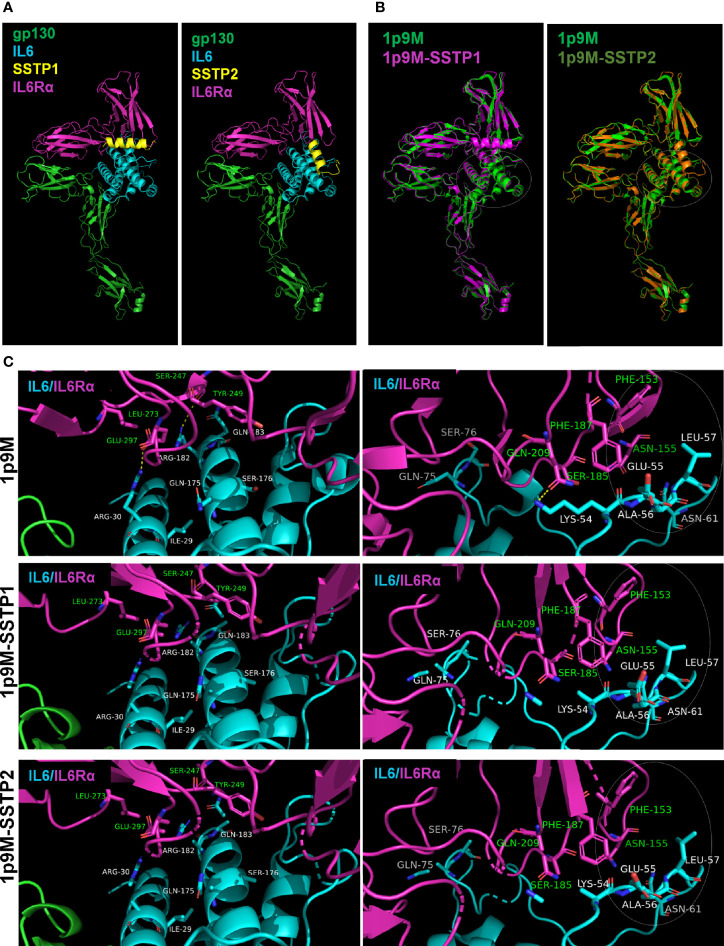
Binding of SSTP1 and SSTP2 to IL6/IL6Rα/gp130 complex alters the active site interactions. **(A)** The predicted structure of SSTP1 and SSTP2 was docked to the crystal structure of trimeric complex 1p9M, and the stable structures identified were obtained after dynamic simulation. **(B)** The SSTP1 or SSTP2-bound active trimeric complex was superimposed to 1p9M. The white circle represents the site with unique changes. **(C)** High resolution images of the interacting residues of IL6 and IL6Rα. The residues of IL6 chain is marked using white font color and the green font represents the residues of IL6Rα. The circle with dotted line denotes the site with unique changes.

To get insight into dynamics of peptide binding, we performed some *in silico* analyses. As the peptides’ crystal structures were not known, we used the 3D structure of another peptide from the same family, Temporin-1 Ta, as a template to predict the structures of SSTP1 and SSTP2 (**Supplementary Figure 4**). Since signaling depends on the formation of the active complex of IL6 and its receptors, we docked the peptides to the reported crystal structure of the active hexameric complex. The analysis predicted stronger interaction for SSTP1 than SSTP2 (with scores -985.038 and -789.990 for SSTP1 and SSTP2, respectively). Dynamic simulations for the structures were performed, and the resulting stable conformations with the least energy were analyzed further. Both SSTP1 and SSTP2 bind to the interface between IL6 and IL6Rα, but in a different orientation (**Figure 6A**).

The superimposition of the peptide-bound complexes to the unbound complex showed that a unique rearrangement occurs in the IL6/IL6R interface of SSTP1-bound complex, specifically in a region that forms an interface between gp130 of the other trimer (**Figure 6B**). The hydrogen bonds formed between Arg30 and Glu297; Arg182 and Ser247; as well as Lys54 and Gln209 were disrupted when SSTP1 or SSTP2 binds to the complex (**Figure 6C**). On further analysis, it was found that the significant unique change observed in the SSTP1-bound complex is in the interface region constituted by Phe153 and Asn155 of IL6Rα and Lys54, Glu55, Ala56, Leu57 and Asn61 of IL6 (**Figure 6C**). There was a difference in the values for accessible surface area, buried surface area and solvation energy for these residues in SSTP1-bound 1P9M in comparison to 1P9M and SSTP2-bound 1P9M, so that the conformation of these residues was significantly altered.

### SSTP1 Down-Regulates JAK/STAT and MAPK Pathways Activating JNK/AP1 Pathway

**Figure 7:**
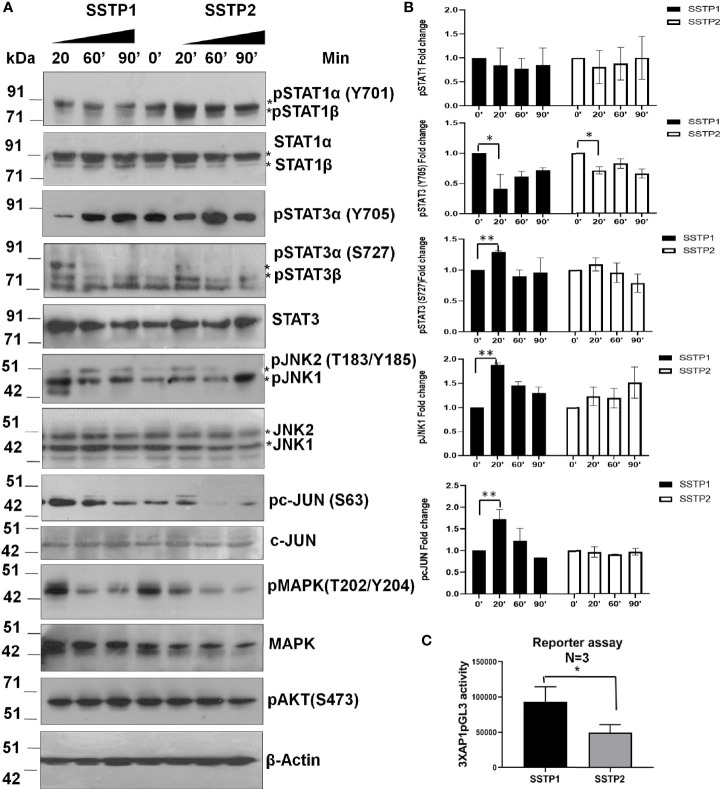
SSTP1 preferentially activates JNK/AP1 pathway **(A)** HSC-4 cells were treated with the peptides as indicated before for the indicated time points, and the lysates were used for western blot. **(B)** Densitometric analysis was performed and relative phosphorylation of each molecule was calculated. Fold change based on untreated from biological duplicates were plotted. **(C)** A luciferase reporter assay was performed using 3×AP1pGL3 reporter construct after treatment with the peptides (10 μM) for 48h. The beetle luciferase activity was normalized with renilla luciferase activity. *p < .05, **p < .01.

There are evidences to show that the IL6/IL6R pathway leads to the activation of JAK/STAT, JNK/AP1, MAPK and PI3K/NF-κB pathways (28–31). The phosphorylated forms of STAT1 (Y701) and STAT3 (Y705) were probed to check the JAK/STAT pathway activation. Another phosphorylation of STAT3 at S727, which is reported to be independent of JAK activation (32), was also probed. Even though SSTP1 did not affect STAT1α (Y701) phosphorylation, it drastically reduced STAT3 (Y705) phosphorylation without changing pSTAT3 (S727) levels. On the other hand, SSTP2-treatment did not significantly alter the phosphorylations of STAT1α or STAT3 (**Figures 7A, B**). SSTP1 markedly up-regulated phosphorylation of JNK1 and pcJUN compared to SSTP2. Though both the peptides down-regulated pMAPK levels, pAKT levels were unaffected. All these biochemical analyses point out that SSTP1 preferentially down-regulates JAK-mediated-phosphorylation of STATs and MAPK, simultaneously up-regulating phosphorylation of JNK1 and c-JUN. A luciferase AP1 reporter assay showed that AP1 is activated by SSTP1 in comparison to SSTP2 (**Figure 7C**).

### SSTP1-Mediated Induction of JNK/AP1 Pathway Depends on IL6/IL6Rα/gp130 Complex

**Figure 8:**
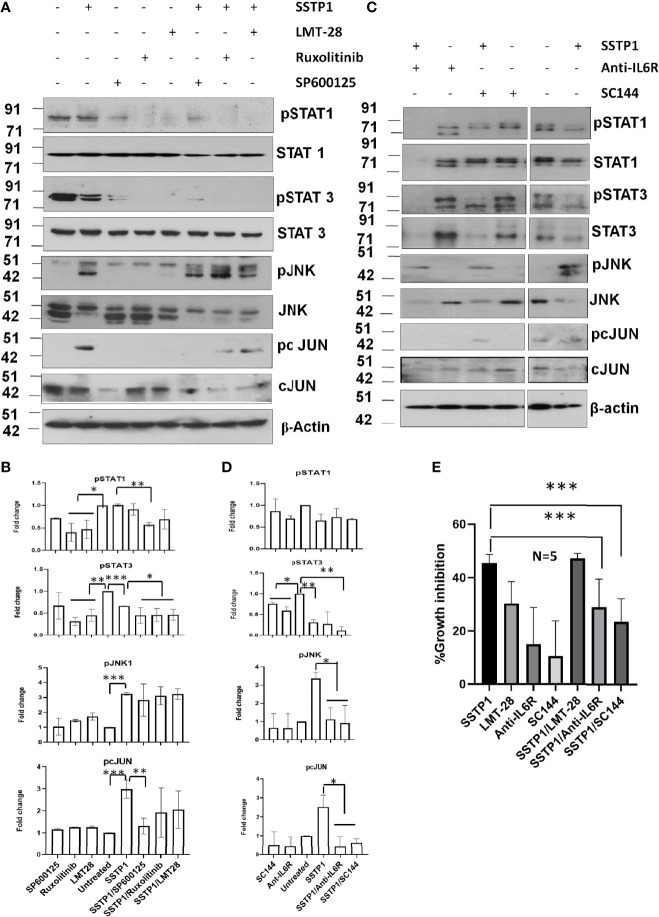
SSTP1-induced pcJUN activation depends on JNK and on gp130 and IL6Rα. **(A)** HSC-4 cells treated with SSTP1 (30 minute) and the indicated inhibitors were used for western blot analysis of the indicated molecules **(B)** Densitometric analysis was done for the bands of **(a)** and relative phosphorylation based on untreated is plotted. **(C)** The cells treated with SSTP1 (30 minutes) and IL6R**α** antibody or SC144 were used for western blot analysis of the indicated molecules. **(D)** Densitometric analysis was done for the bands of **(C)** and relative phosphorylation based on untreated is plotted. **(E)** Growth inhibition analyzed by MTT assay after 4h treatment with SSTP1(10 μM) with or without the inhibitors. LMT-28 (60 μM). For all the experiments SSTP1 (10 μM) was used with or without LMT-28 (60 μM), Ruxolitinib (30 μM) or SP600125 (20 μM), Anti-IL6Rα (10µg/ml) or SC144 (40 μM). Cells were pre-treated with the inhibitors LMT-28 (1.5 h), Ruxolitinib (1.5h) or SP600125 (1h), Anti-IL6Rα (2h) or SC144 (2h) prior to the addition of SSTP1. *p < .05, **p < .01, ***p < .001.

**Figure 9:**
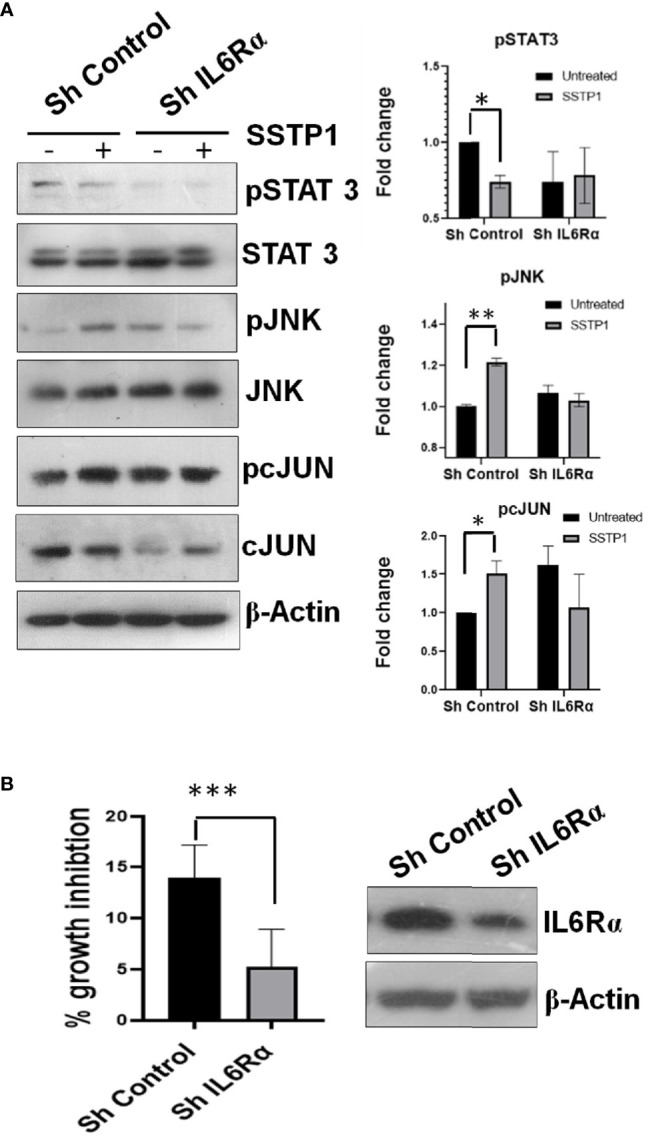
Knock-down of IL6Rα abolishes the apoptotic activity of SSTP1. **(A)** HSC-4 cells stably expressing either control shRNA or IL6Rα shRNA were treated with SSTP1(10 μM) for 30 minutes and western blot was performed for the indicated molecules. The relative phosphorylation based on untreated control ShRNA is plotted. **(B)** The control and IL6Rα- knocked-down cells were treated with SSTP1 for 4h and MTT was performed. The percentage growth inhibition based on the untreated cells is plotted. The western blot showing the knock-down is also shown. *p < .05, **p < .01, ***p < .001.

Inhibitors for IL6 (LMT-28), JAK (S-Ruxolitinib) and JNK (SP600125) were used to check the dispensability of each of these molecules in activating cJUN by SSTP1. The addition of these inhibitors blocked the phosphorylation of STAT1 and STAT3 (**Figures 8A, B**). The SSTP1-mediated up-regulation of pJNK was not appreciably affected by either of these inhibitors, while pcJUN up-regulation was inhibited by only SP600125 (**Figures 8A, B**). At the same time, SC-144, the inhibitor for gp130, and an antibody for IL6Rα significantly reduced phosphorylation of STATs, JNK and cJUN (**Figures 8C, D**). The SSTP1-induced growth inhibition of cancer cells was significantly inhibited by anti-IL6Rα and SC144, whereas LMT28 was ineffective (**Figure 8E**). To further confirm the dependence of SSTP1 activity on IL6Rα, we used lentiviral knock-down of IL6Rα in HSC-4 cells. SSTP1 treatment did not induce down-regulation of STAT3 phosphorylation or up-regulation of pJNK or pcJUN in IL6Rα-knocked-down cells (**Figure 9A**). Moreover, SSTP1-induced growth inhibitory activity was impaired in cells stably expressing IL6Rα shRNA (**Figure 9B**).

### Growth Inhibition by SSTP1 Depends on IL6Rα Levels

**Figure 10:**
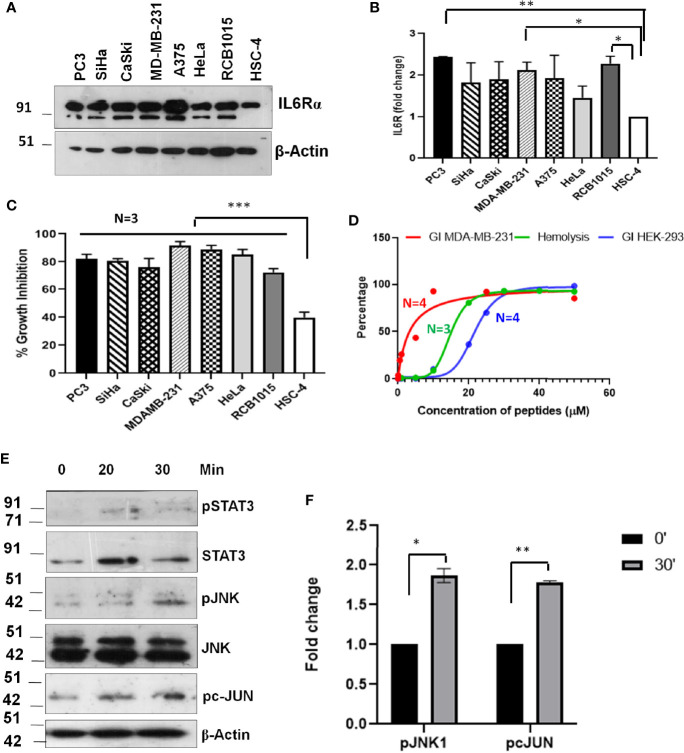
Sensitivity of cancer cells to SSTP1 depends on the level of IL6Rα. **(A)** Western blot performed using cell lysates of different cancer cells. **(B)** The densitometric analysis of the bands of the blot using ImageJ. **(C)** Growth inhibition analyzed by MTT assay using 10 μM SSTP1 treated for 48h. **(D)** Growth inhibitory curve of SSTP1 for MDA-MB-231 cells and HEK-293 cells analyzed after 48h treatment with SSTP1 and Hemolysis induced by SSTP1 on human RBC. The error bars indicate SEM of three biological replicates **(E)** MDA-MB-231 cells were treated with 4.5 μM SSTP1 for the indicated time intervals and probed for the indicated molecules by western blot. **(F)** The blots were quantified using ImageJ, and the graphs were plotted. *p < .05, **p < .01, ***p < .001.

The evaluation of the mechanism of induction of growth inhibition by SSTP1 showed that it depends on the modulation of IL6/IL6R pathway by binding to IL6Rα. This suggested that the peptide might have better growth inhibitory property on IL6Rα-overexpressing cell lines. Since triple-negative breast cancer, prostate cancer and lung cancer express high level of this molecule, we investigated the growth inhibitory property of SSTP1 on these cell lines. The up-regulation of IL6Rα expression was confirmed (**Figures 10A, B**). As we expected, there was a drastic increase in the growth inhibitory potential for SSTP1 in these cells (**Figure 10C**). Notably, there was 91% growth inhibition for triple-negative breast cancer cell line MDA-MB-231. At the IC_50_ concentration (4.5 μM) of this cell line, SSTP1 induced activation of JNK and phosphorylation of c-JUN, while the level of pSTAT3 was extremely low in treated and untreated cells (**Figures 10D–F**). Although SSTP1 induced hemolysis, as many of the bioactive peptides, SSTP1-induced hemolysis at 5 μM was only-0.25% (**Figure 10D**). Further, an epithelial normal cell, HEK 293, was also used to test the sensitivity to SSTP1. The IC_50_ concentration was calculated to be 21. 72 μM.

## Discussion

Temporins are a class of HDPs with antibacterial and antitumor activities, reported from a wide range of frogs of the Ranidae family (33). A few of the family members like Temporin-1CEa, TemporinA, and Temporin-SHa are shown to have antitumor activities against certain cancer types (34–36). The selective cytotoxicity of TemporinA towards lung cancer cells is attributed to the specific phospholipid composition of lung cancer cells (34). The anti-cancer property of Temporin-1CEa depends on both membranolytic activity and mitochondrial pathway of apoptosis (36). Peptides, like temporins with high hydrophobicity, mild amphipathicity, and helical structure, are reported to have antitumor activities (37). While SSTP1 satisfies these conditions, SSTP2 had no amphipathicity as four of the hydrophobic Leucine residues were replaced by positively charged Arginine, serving as a negative control for SSTP1. As reported for other temporins, SSTP1 also induced apoptosis by mitochondrial pathway (35, 36). Even though induction of apoptosis by HDPs is known, their upstream signaling pathways and the molecular targets for most of them are not evaluated yet.

The differential expression of various cytokines and their intermediates in our RNA-Seq analysis substantiated their role in immunomodulation, which is required for host defense peptides. The suppressor of cytokine (SOCS) are important negative regulators of JAK-dependent cytokine signaling (38). The SSTP1-dependent up-regulation of *SOCS1* clearly indicated the suppression of cytokine-mediated JAK/STAT signaling. Our pathway analysis suggested the activation of the cytokine signaling pathway, possibly downstream of IL6Rα. This is in accordance with our earlier observation that the peptide SSTP1 appears as speckles inside the cell membrane, which are distinct from transferrin containing clathrin-dependent endosomes, since IL6Rα is known to be internalized through clathrin-independent pathway (39). Further, we also confirmed the physical interaction of the peptide with IL6Rα, reinforcing the importance of IL6/IL6R signaling pathway in SSTP1-induced apoptosis. Although immune activation by chemotaxis and cytokine release are hall marks of HDPs (10), certain temporins are reported to have anti-inflammatory roles by the modulation of cytokine pathway (40, 41). In pseudomonas-infected bronchial cells, TemporinB induces pro-inflammatory responses, while its analogue induces anti-inflammatory responses (42). L-K6, an analog of temporin-1CEb, suppresses the cytokine signaling pathways in LPS-stimulated macrophages (41). In an altered context, like cancer, the peptide induces apoptosis, probably utilizing similar signaling pathways.

Upon binding to IL6Rα and gp130, IL6 activates different signaling pathways like JAK/STAT, PI3K/NF-κB, Ras/MAPK and JNK/AP1 pathways, depending on the context (28–31). Consistent with the up-regulated *SOCS1*, we did not observe the classical proliferative JAK/STAT downstream molecules like *CyclinD1*, *cMYC* and *PIM* (43, 44). We observed an up-regulation of DUSPs in our RNA-Seq analysis, which are the negative regulators for MAPK signaling (45, 46). Another up-regulated molecule was an E3 ubiquitin ligase, *KLHL21*, which is reported to block NF-κB activity by targeting IKKβ (47). JNK/AP1 pathway is generally a survival pathway, but in certain instances it is shown to induce apoptosis (48, 49). Thus the RNA-Seq results suggested that SSTP1 preferentially up-regulates JNK/AP1 pathway by blocking other arms of IL6/IL6R signaling to facilitate apoptosis. The biochemical analysis for the major intermediates of STATs, JNK, MAPK and PI3K pathways revealed the specific activation of JNK/AP1 pathway, consistent with our prediction from RNA-Seq results. The abrogation of the SSTP1-mediated c-Jun phosphorylation and cytotoxicity upon inhibition of IL6Rα and gp130 strengthened our argument that the AP1 activation depends on the binding of SSTP1 to the active IL6/IL6Rα/gp130 complex.

Among the inhibitors used, LMT-28 blocks the interaction of IL6 with gp130 in the IL6/IL6Rα/gp130 complex (50). S-Ruxolitinib blocks the JAK-mediated phosphorylation of substrates like STATs, and SP60012 blocks the substrate phosphorylation of JNK (28, 51, 52). SC144 is the inhibitor for gp130 and an antibody to the extracellular domain of IL6Rα was used to block the IL6Rα activity. All these inhibitors are considered as IL6 blockers as all of them ultimately blocks STAT activation. Our results showed that none of these molecules can activate AP1 pathway. At the same time, SSTP1 effectively induces cytotoxicity and apoptosis by the activation of IL6R/JNK/AP1 pathway with concomitant down-regulation of IL6-mediated STAT and MAPK signaling, which are pro-tumorigenic pathways (50, 53, 54). Thus it is evident that the mechanism of SSTP1-induced cell death is different from the conventional IL6 blockers that inhibit STAT activation alone.

Binding of IL6 to IL6Rα leads to their interaction with gp130, forming a trimeric complex, which in turn dimerize to form a hexamer (55). In this conformation, gp130 interacts with the IL6/IL6Rα interface of both trimers (55). The conformational change occurring in the extracellular domain of gp130 leads to the autophosphorylation of gp130, and signal transduction through its cytoplasmic domain. Though both the peptides can bind to the active receptor complex, docking analysis predicted a weaker interaction of SSTP2 compared to SSTP1, which was verified by the absence of co-localization in our imaging, and lesser bound peptide in the pull-down experiments. SSTP1 binds to the active receptor complex between IL6 and IL6Rα, significantly rearranging the active site residues that influence downstream signaling. Detailed analysis of the IL6/IL6Rα interface residues in the peptide-bound and unbound active receptor complex showed a unique rearrangement of residues. The conformations of residues Lys54, Glu55, Ala56 and Leu57 are altered differentially when SSTP1 or SSTP2 binds to the complex, to give a unique conformation for SSTP1-bound active receptor complex. Among these residues, Lys54, Glu55 and Leu57 are reported to have an essential function in IL6 dependent activities (56). Lys54 and Glu55 are critical for the binding of IL6 to IL6Rα, while Leu57 is required for its binding to gp130 of the other trimer (56). When these residues were mutated, IL6-dependent proliferative activity of A375 cells was shown to be reduced, wherein mutation of Leu57 had the maximum effect (56). So our results show that the residues interacting at the interface of IL6 and gp130 from the other trimer are altered upon SSTP1 binding, which might alter the conformational changes induced in gp130 strand upon binding to the trimer. This change in gp130 could further alter the substrate specificity of the downstream signaling. Among the inhibitors used, LMT-28 also binds to the same active receptor complex, but at a different site *i.e*. the IL6/gp130 interface, probably explaining the differential outcome of SSTP1 and LMT-28. Since there is no competitive binding for these two molecules, LMT-28 fails to block the activity of SSTP1, as we observed in our analyses.

In MDA-MB-231 cells, SSTP1-induced cell death primarily depending on the activation of JNK, implying the SSTP1-induced JNK activation is sufficient to induce cell death in IL6Rα-overexpressing cancer cells. Amphipathic peptides exhibit membranolytic activity, which increases proportionally to the concentration. As we have shown, the membranolytic activity of the peptide on RBC is low at the IC_50_ concentration of MDA-MB-231 cells. Moreover, the cytotoxicity induced by SSTP1 on normal nucleated epithelial cells, like HEK-293, which could result from the membranolytic activity of SSTP1, was also lower at the IC_50_ concentration of MDA-MB-231 cells. Hence we postulate that when cells express high levels of IL6Rα, even low amounts of SSTP1that do not lead to membranolytic activity is sufficient to bind to the receptor, and act preferentially through the JNK-dependent mechanism for inducing apoptosis. Thus, identification of the target of the HDPs helps to identify the sensitive cell types, and achieve the antitumor activity without eliciting the nonspecific side effects. Our study emphasizes the potential of antitumor HDPs with specific membrane targets for clinical translations, since it induce target specific apoptosis, alleviating the side effects.

## Data Availability Statement

The datasets presented in this study can be found in online repositories. The names of the repository/repositories and accession number(s) can be found below: https://www.ncbi.nlm.nih.gov/geo/, GSE173131.

## Ethics Statement

The studies involving human participants were reviewed and approved by Institutional Human Ethics Committee of Rajiv Gandhi Centre for Biotechnology, Thiruvananthapuram. The patients/participants provided their written informed consent to participate in this study.

## Author Contributions

ShG conceived the idea, conducted the experiments, acquired and analyzed the data. SKU, GM, and AM performed the blotting experiments. GS did the reporter assay. SJ did the statistical analysis. VTVK and SaG contributed to the peptide identification. SKC did the docking and simulation analyses. DV did the peptide structure predictions. SRCN and SNS did the RNA-Seq. data analysis, and TTM conceived the idea, coordinated the experiments and prepared the manuscript. All authors contributed to the article and approved the submitted version.

## Funding

SG received financial support from Kerala State Council for Science Technology and Environment, under VK Young Scientist Scheme (717/2014/KSCSTE). AM acknowledges the financial support from University Grant Commission, India (19/06/2016 (1) EU-V/318501). GM acknowledges the funding received from University Grant Commission, India (952/(CSIRUGC NET JUNE2019-368272).

## Conflict of Interest

A patent application has been filed for the peptide, SSTP1 in USA. “Apoptosis Inducing Peptide (SSTP1)”, PCT/IN2021/050371, in the name of Rajiv Gandhi Centre for Biotechnology, with TM & SG as Investigators.

The remaining authors declare that the research was conducted in the absence of any commercial or financial relationships that could be construed as a potential conflict of interest.

## Publisher’s Note

All claims expressed in this article are solely those of the authors and do not necessarily represent those of their affiliated organizations, or those of the publisher, the editors and the reviewers. Any product that may be evaluated in this article, or claim that may be made by its manufacturer, is not guaranteed or endorsed by the publisher.
