# Supplementary material for: SSTP1, a Host Defense Peptide, Exploits the Immunomodulatory IL6 Pathway to Induce Apoptosis in Cancer Cells
Source: Front Immunol. 2021 Nov 19;12:740620. doi: 10.3389/fimmu.2021.740620 (PMC8639500; doi:10.3389/fimmu.2021.740620)
Supplement: Supplementary file 1 [file DataSheet_1.pdf]

SSTP1

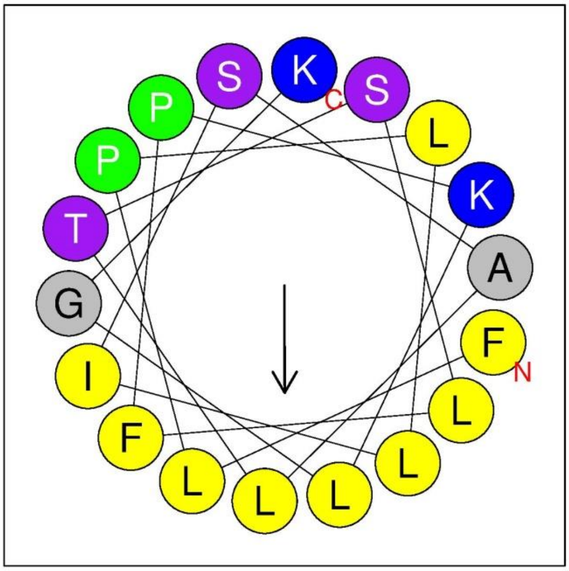

SSTP2

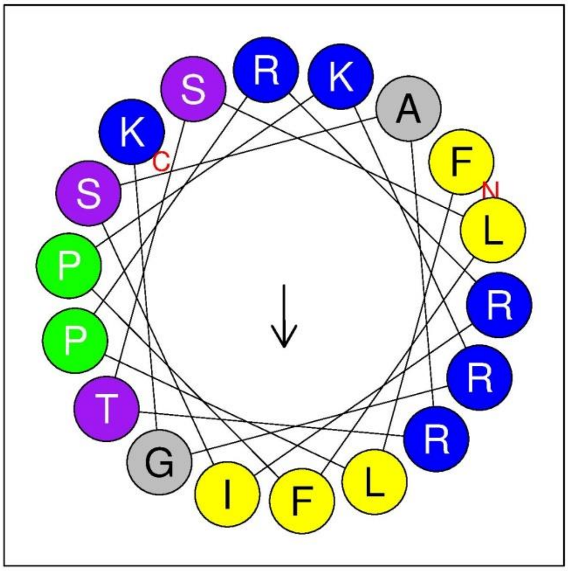

Supplementary Figure 1. Physicochemical characteristics of SSTP1 and SSTP2 Helical wheel projection of the peptides predicted by HeliQuest. The yellow residues indicates the hydrophobic amino acids

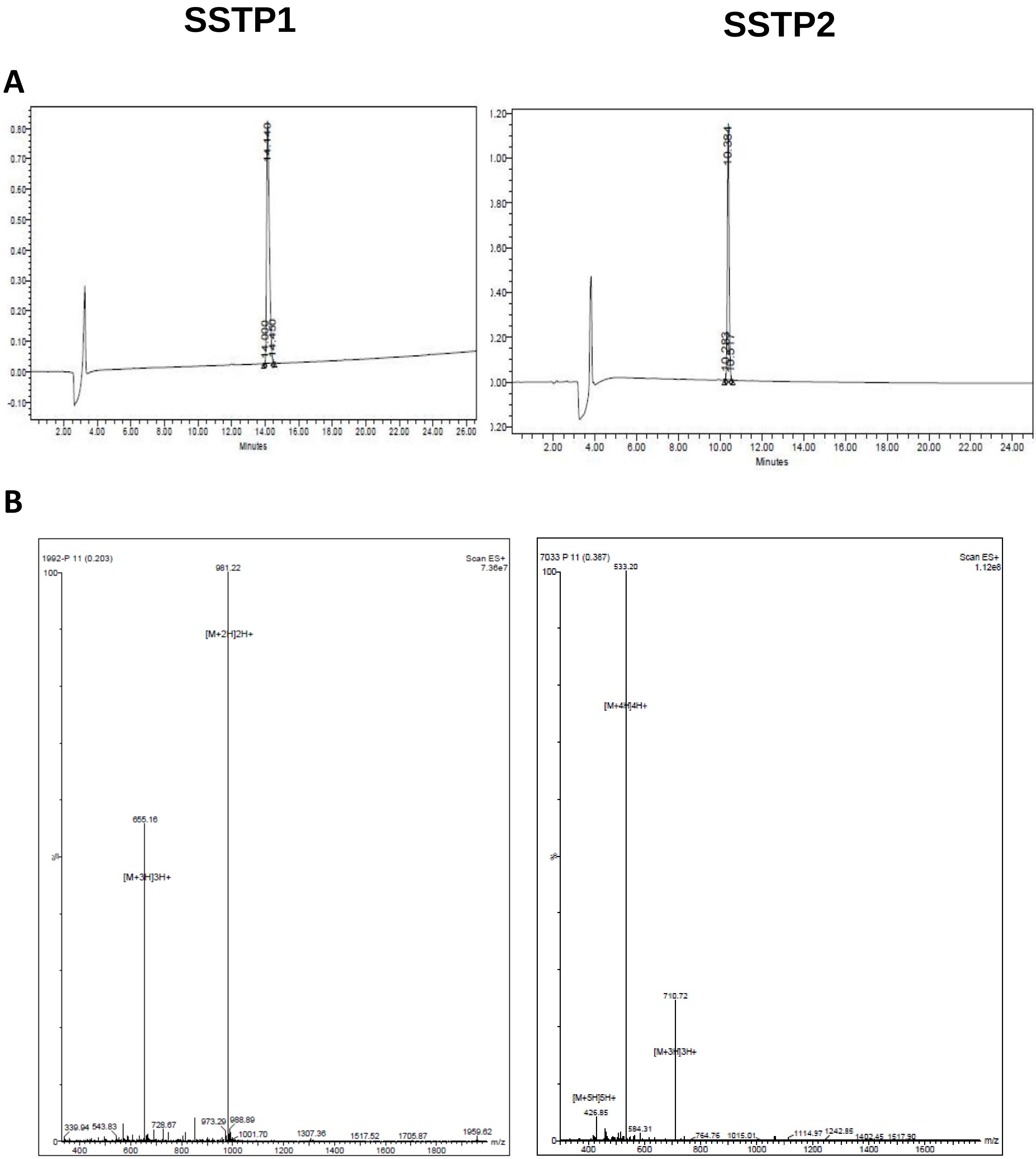

Supplementary Figure 2. confirming the purity of SSTP1 and SSTP2.(A) HPLC profile showing single peak (B) ESI/MS data . SSTP1 shows doubly and triply charged peptide while SSTP2 gives, triply charges and quaternary charged peptide

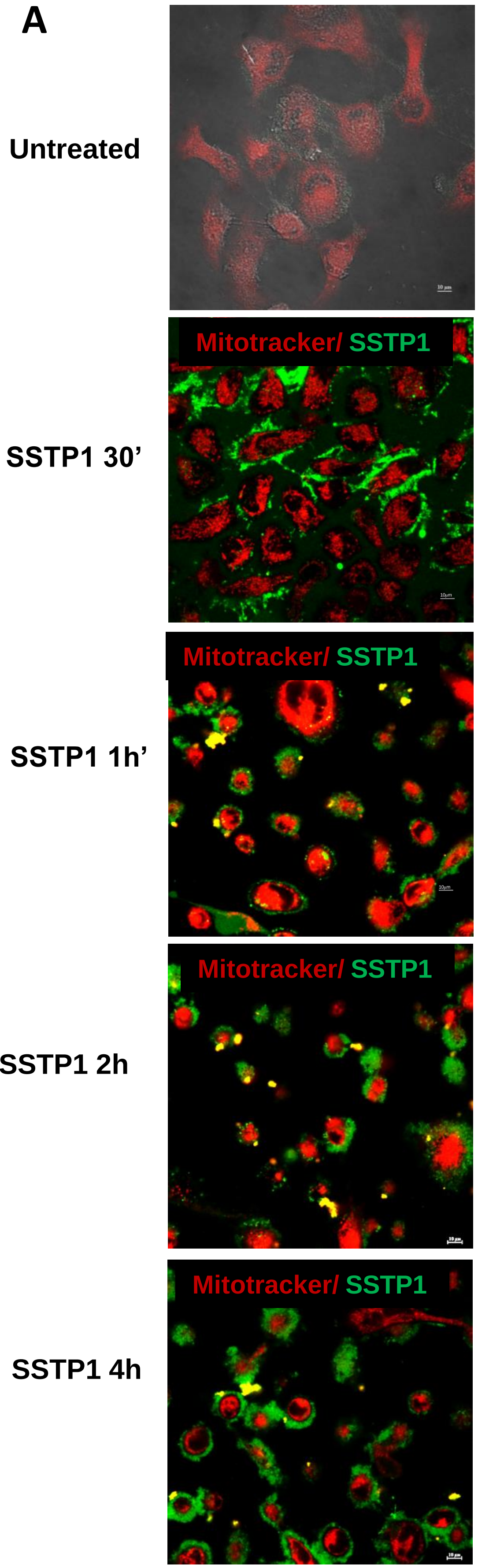

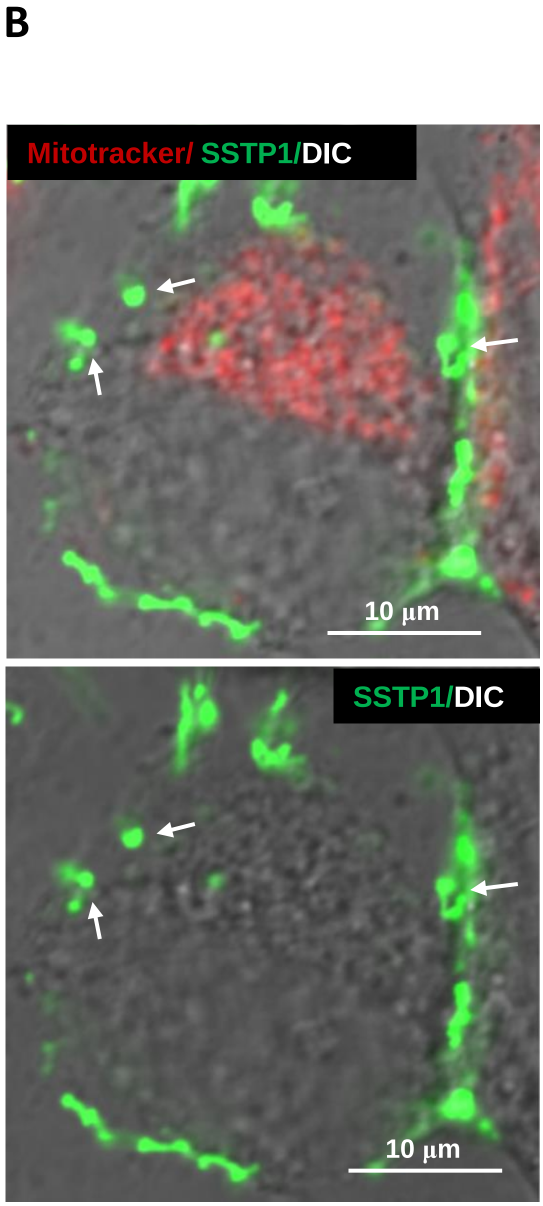

Supplementary Figure 3. Time dependent uptake of SSTP1.(A) The cells were treated with SSTP1-Alexa Fluor 488 for the indicated time interval. Excess peptide was washed off, and the images were acquired. (B) Zoomed images of SSTP1 30 min shown in Fig 2A left panel. The arrow marks indicate the internalization of peptides

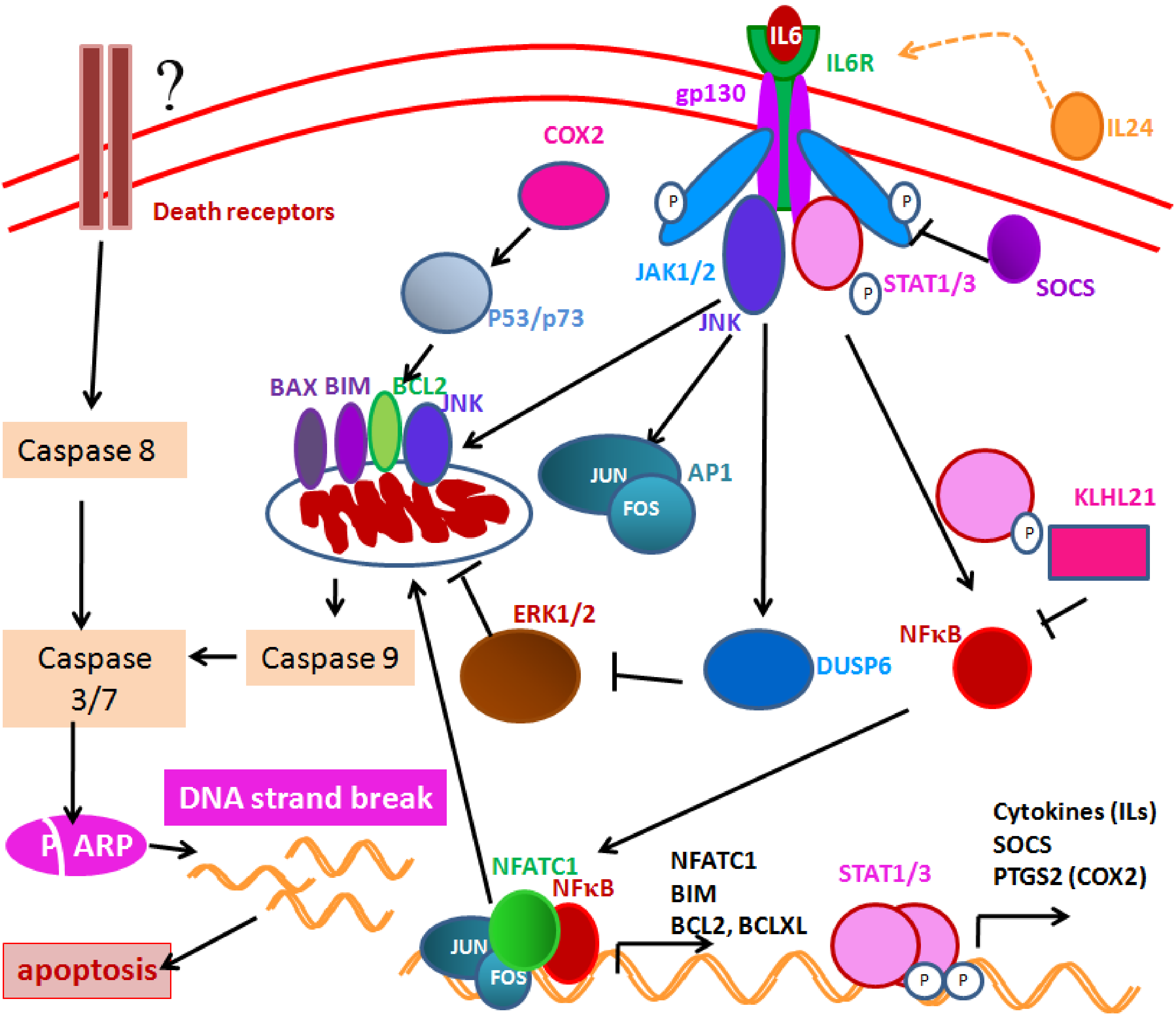

Supplementary Figure 4. IL6 pathway and apoptosis. The diagram represents different downstream signaling molecules and their cross-talk.

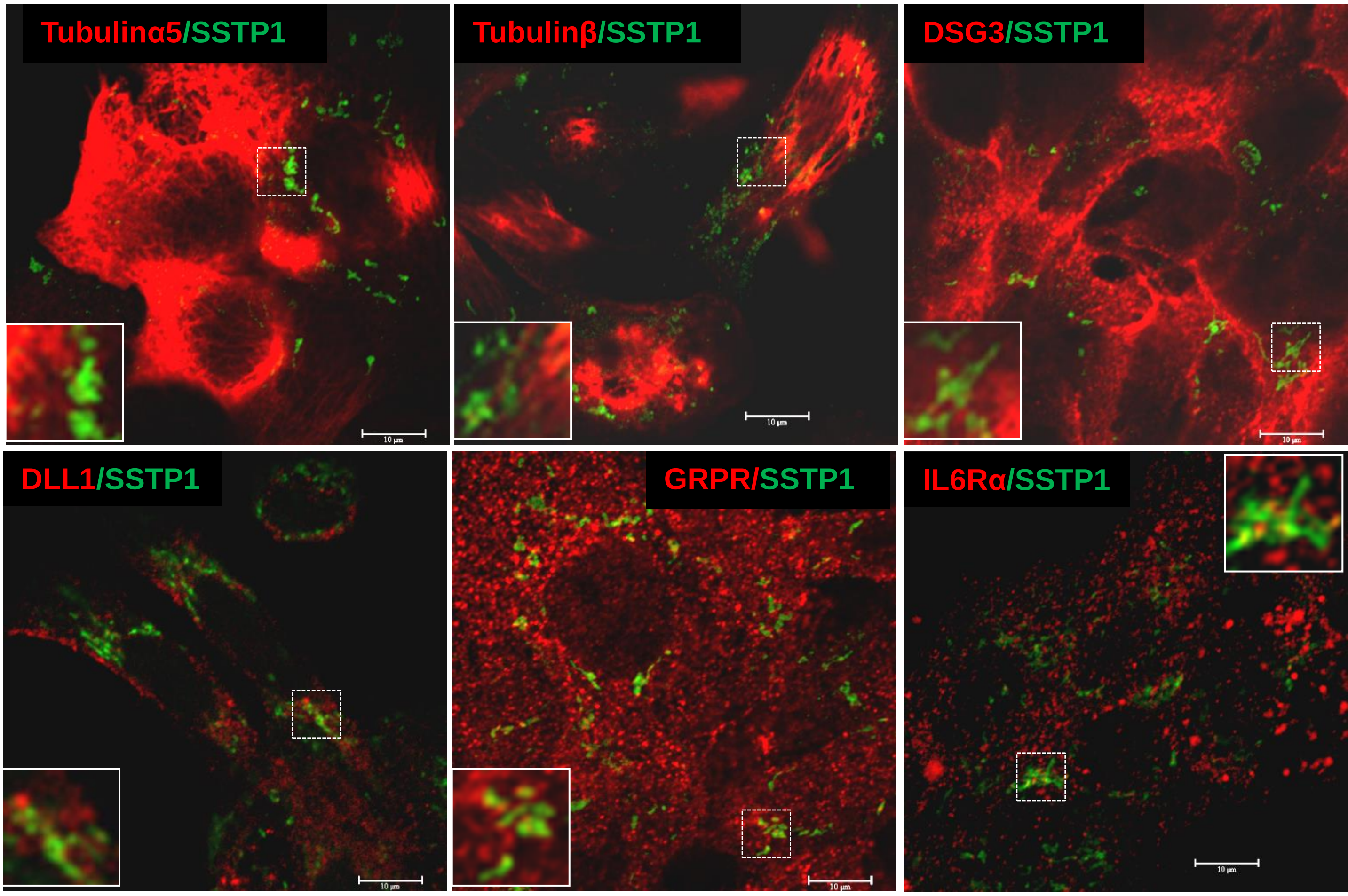

Supplementary Figure 5. SSTP1 interacts specifically with IL6α. Live HSC-4 cells were incubated with SSTP1 labeled with biotin at 37 ºC for 30min. The cells were fixed and stained with indicated antibodies. Streptavidin-488 was used to visualize SSTP1. The inset shows the higher magnification of the dotted square.

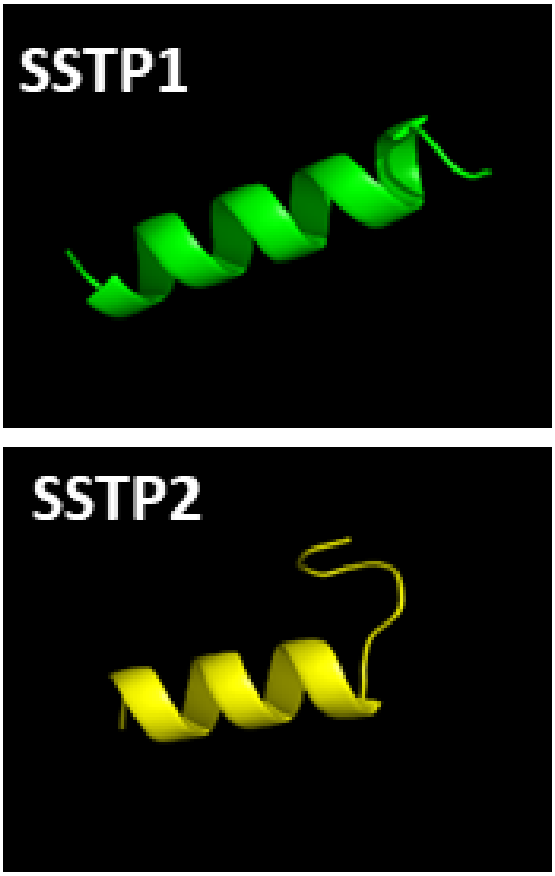

Supplementary Figure 6. The predicted 3D structure of SSTP1 and SSTP2.

Sl.No.	Name of Peptides	No. of amino acids	Mol.Wt. (Da)
1	Brevinin 2IDau1	29	2848.442
2	Brevinin 2IDau2	29	2919.56
3	Brevinin 2IDau3	29	2876.499
4	Brevinin 2IDau4	30	3115.735
5	Brevinin 2IDau5	29	3005.669
6	Brevinin 1 IDau5	24	2513.234
7	Temporin1IDau1	18	1958
8	Temporin 1IDau 2	14	1475.925
9	Temporin 1IDau3	18	1930.429
10	Gastrin IDau	34	3891.389
11	SpinulosainIDau	26	2830.373
13	Aurin like IDau	18	1800.303
14	Hainanenin like IDau	20	2246.742

Supplementary Table 1. List of peptides identified from skin secretion of *Indosylvirana aurantiaca*

**Physico Chemical properties**	**SSTP1**	**SSTP2**
Length	18 a.a	18a.a
Molecular weight	1958.455	2129.57
Total hydrophobic ratio	55%	33%
GRAVY (i.e. the Grand Average hydropathy value of the peptide)	1.16666666667	0.677777777778
The Wimley-White whole-residue hydrophobicity of the peptide	-2.47 kcal/mol	3.01 kcal/mol
Protein-binding Potential (Boman index	-1.26 kcal/mol	3.14 kcal/mol
Net charge	+2	+7

Supplementary Table 2. Physico chemical parameters predicted by ProtParam

Sl no	User ID	Ensembl Gene ID	Symbol	functional Category
1	TXNIP	ENSG00000265972	TXNIP	Positive regulation of cellular metabolic process
2	RNF223	ENSG00000237330	RNF223	Positive regulation of metabolic process
3	ZDHHC18	ENSG00000204160	ZDHHC18	Positive regulation of macromolecule metabolic process
4	LAMB3	ENSG00000196878	LAMB3	Positive regulation of nitrogen compound metabolic process
5	CD55	ENSG00000196352	CD55	Protein phosphorylation
6	GJB3	ENSG00000188910	GJB3	Regulation of cellular protein metabolic process
7	JUN	ENSG00000177606	JUN	Regulation of protein metabolic process
8	SFN	ENSG00000175793	SFN	Response to lipid
9	PLK3	ENSG00000173846	PLK3	Intracellular signal transduction
10	MFSD2A	ENSG00000168389	MFSD2A	Regulation of protein modification process
11	IL24	ENSG00000162892	IL24	Regulation of intracellular signal transduction
12	IER5	ENSG00000162783	IER5	Regulation of signal transduction
13	ATF3	ENSG00000162772	ATF3	Regulation of phosphate metabolic process
14	KLHL21	ENSG00000162413	KLHL21	Regulation of phosphorus metabolic process
15	IL6R	ENSG00000160712	IL6R	Phosphorylation
16	PPFIA4	ENSG00000143847	PPFIA4	Regulation of protein phosphorylation
17	ABL2	ENSG00000143322	ABL2	Regulation of phosphorylation
18	CYR61	ENSG00000142871	CYR61	Positive regulation of cellular protein metabolic process
19	EPHA2	ENSG00000142627	EPHA2	Cellular response to chemical stimulus
20	BCAR3	ENSG00000137936	BCAR3	Response to oxygen-containing compound
21	KCNK1	ENSG00000135750	KCNK1	Regulation of cell communication
22	CDKN2C	ENSG00000123080	CDKN2C	Regulation of molecular function
23	ID3	ENSG00000117318	ID3	Regulation of signaling
24	ARHGEF2	ENSG00000116584	ARHGEF2	Regulation of response to stimulus
25	DVL1	ENSG00000107404	DVL1	System development
26	RASAL2	ENSG00000075391	RASAL2	Response to organic substance
27	PTGS2	ENSG00000073756	PTGS2	Animal organ development

28	LINC00707	ENSG00000238266	LINC00707	Positive regulation of protein metabolic process
29	FZD8	ENSG00000177283	FZD8	Cell proliferation
30	ARID5B	ENSG00000150347	ARID5B	Regulation of multicellular organismal process
31	DUSP5	ENSG00000138166	DUSP5	Positive regulation of developmental process
32	PPP1R3C	ENSG00000119938	PPP1R3C	Cell migration
33	GPAM	ENSG00000119927	GPAM	Regulation of cell proliferation
34	PPIF	ENSG00000108179	PPIF	Regulation of cell motility
35	DKK1	ENSG00000107984	DKK1	Phosphate-containing compound metabolic process
36	KLF6	ENSG00000067082	KLF6	Cellular response to organic substance
37	VIM	ENSG00000026025	VIM	Phosphorus metabolic process
38	FOSL1	ENSG00000175592	FOSL1	Tissue development
39	MYEOV	ENSG00000172927	MYEOV	Positive regulation of protein modification process
40	ABTB2	ENSG00000166016	ABTB2	Cell motility
41	CDC42EP2	ENSG00000149798	CDC42EP2	Localization of cell
42	SESN3	ENSG00000149212	SESN3	Cell death
43	ETS1	ENSG00000134954	ETS1	Programmed cell death
44	MICAL2	ENSG00000133816	MICAL2	Cell surface receptor signaling pathway
45	ARNTL	ENSG00000133794	ARNTL	Regulation of cell migration
46	KRT6B	ENSG00000185479	KRT6B	Locomotion
47	KRT6C	ENSG00000170465	KRT6C	Regulation of cell differentiation
48	HSPB8	ENSG00000152137	HSPB8	Regulation of locomotion
49	EMP1	ENSG00000134531	EMP1	Regulation of developmental process
50	NR4A1	ENSG00000123358	NR4A1	MAPK cascade
51	PTHLH	ENSG00000087494	PTHLH	Apoptotic process
52	NAV3	ENSG00000067798	NAV3	Signal transduction by protein phosphorylation
53	TMCC3	ENSG00000057704	TMCC3	Regulation of multicellular organismal development
54	LINC00346	ENSG00000255874	LINC00346	Regulation of cellular component movement
55	SPRY2	ENSG00000136158	SPRY2	Cell differentiation

56	STARD13	ENSG00000133121	STARD13	Regulation of programmed cell death
57	CCNA1	ENSG00000133101	CCNA1	Positive regulation of protein phosphorylation
58	RGCC	ENSG00000102760	RGCC	Positive regulation of gene expression
59	LINC01550	ENSG00000246223	LINC01550	Circulatory system development
60	RN7SL2	ENSG00000274012	RN7SL2	Regulation of apoptotic process
61	ZFP36L1	ENSG00000185650	ZFP36L1	Regulation of cell death
62	FOS	ENSG00000170345	FOS	Cellular developmental process
63	PLEKHG3	ENSG00000126822	PLEKHG3	Positive regulation of phosphorylation
64	SAMD4A	ENSG00000020577	SAMD4A	Negative regulation of cell communication
65	AEN	ENSG00000181026	AEN	Negative regulation of signaling
66	ARID3B	ENSG00000179361	ARID3B	Movement of cell or subcellular component
67	ARNT2	ENSG00000172379	ARNT2	Positive regulation of phosphorus metabolic process
68	BNC1	ENSG00000169594	BNC1	Positive regulation of phosphate metabolic process
69	RHCG	ENSG00000140519	RHCG	Regulation of biosynthetic process
70	CYP1A1	ENSG00000140465	CYP1A1	Positive regulation of cell proliferation
71	PIF1	ENSG00000140451	PIF1	Regulation of cellular macromolecule biosynthetic process
72	SEMA7A	ENSG00000138623	SEMA7A	Cellular protein modification process
73	SMAD6	ENSG00000137834	SMAD6	Protein modification process
74	BMF	ENSG00000104081	BMF	Response to stress
75	SOCS1	ENSG00000185338	SOCS1	Regulation of cellular biosynthetic process
76	FOXC2	ENSG00000176692	FOXC2	Positive regulation of signal transduction
77	KIFC3	ENSG00000140859	KIFC3	Negative regulation of developmental process
78	TNFRSF12A	ENSG00000006327	TNFRSF12A	Regulation of macromolecule biosynthetic process
79	PRSS22	ENSG00000005001	PRSS22	Cellular response to oxygen-containing compound
80	RN7SL236P	ENSG00000243870	RN7SL236P	Positive regulation of biosynthetic process
81	FAM83G	ENSG00000188522	FAM83G	Positive regulation of cell migration
82	SOCS3	ENSG00000184557	SOCS3	Positive regulation of multicellular organismal process
83	SPHK1	ENSG00000176170	SPHK1	Positive regulation of cellular biosynthetic process

84	TMEM88	ENSG00000167874	TMEM88	Macromolecule modification
85	USP43	ENSG00000154914	USP43	Positive regulation of cell differentiation
86	GRB7	ENSG00000141738	GRB7	Positive regulation of macromolecule biosynthetic process
87	KDM6B	ENSG00000132510	KDM6B	Cellular response to lipid
88	TNS4	ENSG00000131746	TNS4	Positive regulation of cell motility
89	SOX9	ENSG00000125398	SOX9	Positive regulation of RNA metabolic process
90	TRAF4	ENSG00000076604	TRAF4	Positive regulation of response to stimulus
91	MAP2K3	ENSG00000034152	MAP2K3	Positive regulation of cell communication
92	SERPINB2	ENSG00000197632	SERPINB2	Regulation of transcription, DNA-templated
93	PMAIP1	ENSG00000141682	PMAIP1	Negative regulation of signal transduction
94	MYADM	ENSG00000179820	MYADM	Positive regulation of signaling
95	AXL	ENSG00000167601	AXL	Negative regulation of nitrogen compound metabolic process
96	MIDN	ENSG00000167470	MIDN	Positive regulation of cellular component movement
97	PLEKHF1	ENSG00000166289	PLEKHF1	Negative regulation of response to stimulus
98	JUND	ENSG00000130522	JUND	Regulation of gene expression
99	LDLR	ENSG00000130164	LDLR	Negative regulation of cellular metabolic process
100	ZFP36	ENSG00000128016	ZFP36	Regulation of catalytic activity

Supplementary Table 3. List of differentially regulated genes that are involved in the regulation of apoptosis

Gene	**Name of primer**	**Sequence (5'-3')**	**Ampli. size**
RRAD	RRAD FP	GACATTTGGGAGCAGGACGG	220
	RRAD RP	CATCCACCGAGACCTCACGA	
PTGS2	PTGS2 FP	CAAATTGCTGGCAGGGTTGC	256
	PTGS2 RP	GCCGAGGCTTTTCTACCAGA	
NR4A1	NR4A1 FP	TGCCAGCATTATGGTGTCCG	183
	NR4A1 RP	AACTTCCTTCACCATGCCCA	
NFATC2	NFATC2 FP	CATCCCAGTGACTGCATCC	198
	NFATC2 RP	GGCTTGTTTTCCATGTAGCCA	
RHOB	RHOB FP	CCTACGACTACCTCGAGTGC	134
	RHOB RP	TCATAGCACCTTGCAGCAGTT	
IL6R	IL6R FP	GTTTGTGAGTGGGGTCCTCG	194
	IL6R RP	GCGACGCACATGGACACTAT	
JUN	JUN FP	GTCCGAGAGCGGACCTTATG	183
	JUN RP	CTGGATTATCAGGCGCTCCA	
SOCS1	SOCS1 FP	TTCCCCTTCCAGATTTGACCG	251
	SOCS1 RP	TGCTACAACAACCAGGGGGA	
KLF6	KLF6 FP	GGCAACAGACCTGCCTAGAG	122
	KLF6 RP	CTCCCGAGCCAGAATGATTTT	
FOSB	FOSB FP	GGGGCAAGGTGGAACAGTTAT	126
	FOSB RP	CCGCTTGGAGTGTATCAGTCA	
CD55	CD55FP	CTTCCAAGGTCCCACCAACAG	206
	CD55RP	ACGGGTAGTACCTGAAGTGGT	
FOSL1	FOSL1FP	CTGGTGCCAAGCATCAACAC	186
	FOSL1RP	TTCACAAGGCCTTCGACGTA	
BCL11A	BCL11A FP	TAAACTTCTGCACTGGAGGGG	162
	BCL11A RP	CATGCACTGGTGAATGGCTG	
SOX2	SOX2 FP	ATGGACAGTTACGCGCACAT	269
	SOX2 RP	GACTTGACCACCGAACCCAT	
TP73	TP73 FP	CCACAGGTGGGGACGGAAT	207
	TP73 RP	CTCCCGGTAGTGGTCCTCATC	
BMF	BMF FP	CGGGAGCTTGCTCTCTGC	212
	BMF RP	AAAAGAGTCGCTGGGGTTCC	

Supplementary Table 4. List of primers used for Real time PCR
